# Are Tail and Ear Movements Indicators of Emotions in Tail-Docked Pigs in Response to Environmental Enrichment?

**DOI:** 10.3390/ani9070449

**Published:** 2019-07-16

**Authors:** Míriam Marcet-Rius, Emma Fàbrega, Alessandro Cozzi, Cécile Bienboire-Frosini, Estelle Descout, Antonio Velarde, Patrick Pageat

**Affiliations:** 1 Physiological and Behavioral Mechanisms of Adaptation Department, IRSEA (Research Institute in Semiochemistry and Applied Ethology), Quartier Salignan, 84400 Apt, France; 2 Animal Welfare Program, IRTA (Insitute of Agrifood Research and Technology), Centre de Control i Avaluació de Porcí, 17121 Monells, Girona, Spain; 3 Research and Education Board, IRSEA (Research Institute in Semiochemistry and Applied Ethology), Quartier Salignan, 84400 Apt, France; 4 Statistical Analysis Service, IRSEA (Research Institute in Semiochemistry and Applied Ethology), Quartier Salignan, 84400 Apt, France; 5 Semiochemicals’ Identification and Analogs’ Design Department, IRSEA (Research Institute in Semiochemistry and Applied Ethology), Quartier Salignan, 84400 Apt, France

**Keywords:** animal welfare, pig assessment, positive emotions, negative emotions, enrichment material

## Abstract

**Simple Summary:**

The assessment of animal welfare should involve physical and mental welfare. Physical welfare is relatively easy to measure. Nevertheless, there is a lack of feasible indicators of positive emotions in farm animals, which causes difficulties in obtaining a complete analysis of welfare. To improve the quality of life of farm animals, it is necessary to be able to assess their welfare in a valid and feasible way. This study aimed to determine whether environmental enrichment, understood to be positive for animal welfare, could influence tail movement and ear movement in fattening pigs. These indicators could be used to assess emotions, with positive or negative valences, as suggested in previous studies on mini-pigs and pigs. The results showed that tail movement was a valid and feasible indicator of positive emotions; pigs moved their tails a greater number of times when they interacted more with enrichment than when they interacted less with it. Regarding ear movements, this study revealed a need for further investigation. This research could play an important role in improving the analysis of different emotions in pigs, thereby improving the assessment of animal welfare in pig breeding systems using valid, feasible, and noninvasive indicators of emotions.

**Abstract:**

The inclusion of emotional indicators in farm monitoring methods can improve welfare assessments. Studies in controlled conditions have suggested that increased tail movement is an indicator of positive emotions in pigs, while others have proposed that increased ear movements are linked to negative emotions. This study aimed to investigate these indicators in pig farm conditions to analyze their validity and the effect of enrichment on welfare. Thirty-six pigs received one of the following enrichment materials: straw in a rack, wooden logs, or chains. Behavioral observations were performed by focal sampling. The results showed that tail movement duration was significantly higher when pigs exhibited “high use” (three or more pigs in a pen interacting with the enrichment) than when they exhibited “low use” (fewer than three) of enrichment (*p* = 0.04). A positive correlation was found between tail movement frequency and duration (r = 0.88; *p* = 0.02). The increase in tail movement could be considered an indicator of positive emotions in pigs when measured with other categories of indicators. Regarding ear movements, no significant difference was found. Future studies should further investigate these indicators thoroughly, as the results could be useful for improving the assessment of emotions in pigs.

## 1. Introduction

The assessment of farm animal welfare requires a good understanding of the animals’ affective experiences, including their emotions [1]. Emotions are transient reactions to short-term triggering events, and their continued occurrence can cause longer-lasting affective states, which represent good or poor welfare [2]. The inclusion of indicators of emotions in farm monitoring methods can improve welfare assessments beyond the traditional focus on the mere absence of disease and distress [3] and good feeding and housing practices [4].

Currently, animal welfare assessments of farm animals do not always include the assessment of emotions, either positive or negative. In addition, when they are included, they can be influenced by a subjective assessment of the auditor, for example, in the case of the Qualitative Behaviour Assessment [5]. Thus, more objective and feasible indicators of the emotions of farm animals could be very useful for providing new insights into both animal welfare assessments and our understanding of the welfare state, either positive or negative, of farm animals [3].

Emotional experiences are valenced, being perceived as positive or negative, rewarding or punishing, pleasant or unpleasant [6]. Emotional experiences also vary in terms of reported activation or arousal [6]. Emotional arousal can be defined as an emotional activation, in which animals’ bodies experience heightened physiological activity, and extremes of emotion, being positive, such as excitement, or negative, such as anger [7].

Some authors [8,9] have suggested that an increase in tail movement for pigs is related to positive emotions: both tail wagging and changes in tail posture occur more often during rewarding events than during aversive events. Other studies have also reported that increased tail wagging in pigs is related to positive situations such as social greetings [10,11] and play [12]. Recent studies performed under controlled conditions with mini-pigs provided clear results: tail movement duration was significantly higher when animals performed play behaviors when provided with enrichment materials (medium-sized dog toys) than when animals did not play because they were not provided with enrichment materials [13,14]. Play is believed to trigger positive emotions [15,16], which suggests that a long tail movement duration reflects positive emotions in pigs.

In contrast, Reimert and colleagues [8] suggested that increased frequency of ear posture changes tended to be linked to negative situations and were less frequent in positive situations. Marcet-Rius et al. [14] showed that under controlled conditions, ear movement frequency was significantly lower in a group of mini-pigs that was allowed to play with an object than in a group that was not allowed to do so. Thus, the study provided new information about this potential indicator of emotions in pigs by demonstrating that pigs showed fewer ear movements in a positive situation than in a control situation (with no stimulus or enrichment). Another study performed under controlled conditions with mini-pigs showed that ear movement frequency and the frequency and duration of other known indicators of poor welfare (agonistic behavior and displacement behavior) were significantly lower in a group provided with straw than in a control group that was not provided with any manipulatable material or other enrichment [17]. The provision of straw in pig production systems is widely presumed to be beneficial to animal welfare [18,19], and the observation that ear movement frequency was significantly lower in pigs provided with straw than that during the control session, together with two other indicators of poor welfare, strongly suggests that a high frequency of ear movement is more likely to be linked to negative emotions or at least is more common in a poor environment. Similar results regarding ear posture changes have been found in other species, such as sheep [20,21,22], dogs [10], and horses [23].

Intensive production systems are often very barren with concrete (slatted) floors and no substrate in which the animals can root [24]. Such environments hamper the ability of pigs to express some key behaviors, such as exploration and foraging [25]. As a consequence, harmful and manipulative behaviors such as ear and tail biting often occur at high frequencies [24]. Successful enrichment should decrease the incidence of abnormal patterns of behavior and increase the performance of behaviors such as exploration, foraging, play, and social interaction, which are within the range of the animal’s normal, species-specific behavior [25]. Additionally, enrichment could also enhance performance by improving, for example, the feed conversion ratio [26]. Recent studies have shown that pigs have more optimistic judgment biases in enrichment environments, a fact that indicates a more positive affective state and, hence, better welfare [27]. The provision of appropriate environmental enrichment to pigs is mandatory by law in Europe [28]. Additionally, the European Union encourages leaving pigs undocked per the EU Recommendation of 2016 [29]. Tail-docking is a procedure consisting of cutting the tail of pigs, sometimes without anesthesia and analgesia, which has been commonly used to reduce the risk of tail biting. Tail-biting incidents also occur when tails are docked; therefore, docking as such does not solve the tail-biting problem [29]. Thus, the need to identify indicators of positive welfare that could support the use of enrichment materials to reduce the incidence of tail biting is important. Tail movement could be a practical tool for farmers to assess positive animal welfare.

Assuming that increases in tail movement and ear movement are respective indicators of positive and negative emotions in mini-pigs in a controlled system, the aim of the present study was to investigate these potential indicators in pigs provided with enrichment materials at an experimental farm. We hypothesize that increases in tail and ear movements could be indicators of positive and negative emotions in pigs, respectively. In this study, enrichment materials were provided to pigs to allow exploratory behavior, which is very important in these animals [30]. We measured how the interaction with the enrichment material influenced these potential indicators of emotions in pigs.

## 2. Materials and Methods

The housing, husbandry and use of animals for the procedures described in this manuscript were carried out according to Spanish and European legislation. The project, including this experimental procedure, was approved by IRTA’s (Institute of Agrifood Research and Technology, Caldes de Montbui, Spain) Ethics Committee (approval number: AGL2015-68373-C2-2-R).

### 2.1. Animals and Housing

**Figure 1:**
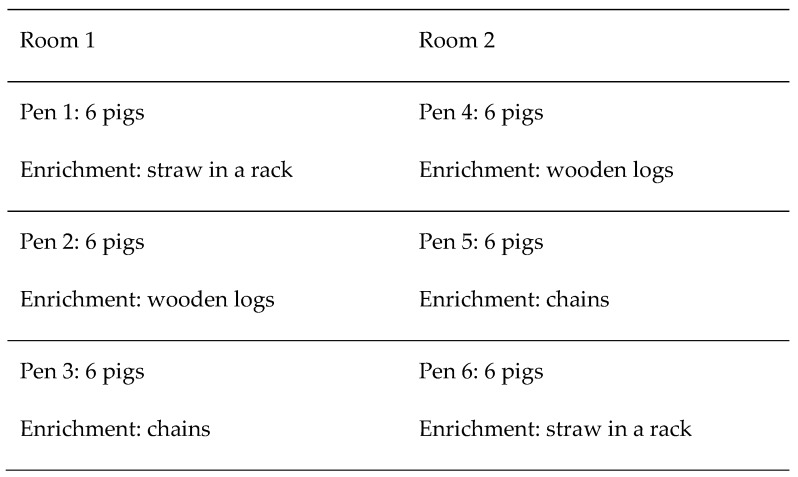
Distribution of different types of enrichment material.

The pigs (*Sus scrofa domesticus*) (n = 36; entire males) involved in this study were a conventional commercial cross between Landrace x Large White dams with a Pietrain sire. All the pigs came from the same farm (Batallé^®^ selecció, Riudarenes, Girona, Spain), and they arrived at the experimental farm at IRTA at two months of age. All of the pigs were tail-docked: the commercial farm of origin was in the process of implementing an action plan (i.e., undertaking correction actions to prevent tail biting if necessary) after carrying out a risk analysis of tail biting as required by the EU Recommendation of 2016 [29]; they will start leaving pigs undocked when the action plan is proven to work, first with docked pigs. Additionally, the results of a preliminary trial conducted on the same research farm by the authors revealed that using some of the single enrichment items used in this study, under similar conditions, led to an increased occurrence of tail biting. Therefore, due to ethical concerns, the authors decided to perform the present study in docked pigs as a first step towards moving to undocked pigs. The pigs entered the present study at 2.5 months of age. Thirty-six pigs were involved in the study and were divided into two identical rooms with 18 pigs per room. In each room, there were three identical pens with six animals in each pen, with a stocking density of 0.9 m^2^/pig. In every pen, each pig had a different colored tag (blue, yellow, red, orange, green, and white) to differentiate it from other pigs. Three different types of enrichment materials were constantly provided to the animals for three months, once for each pen in each room: straw in a rack, wooden logs, or chains. More precisely, there was one pen in each room with each type of enrichment material, totaling two pens for each material (12 pigs for each material, divided into two pens of 6 animals) (Figure 1).

For this experimental procedure, the most important analysis was the interaction or lack of interaction with the enrichment, regardless of type, as a way to create a positive situation for the animals and a control situation (lack of interaction). All the animals were provided enrichment materials, meaning that all of them could interact with the materials and that no animals were in a nonenriched pen. All the pigs were maintained under the same housing conditions and managed in the same way and by the same stock people. The rooms where the pigs were housed and where the experiment was carried out had an automatic control system for regulating temperature (22 ± 5 °C) and ventilation. The pens had a completely slatted floor. The pigs were fed ad libitum with a commercial pig diet. More precisely, a commercial concentrate was provided in a phased feeding regime (15.04% crude protein and 2.321 kcal net energy at mid-fattening). Animals had continuous access to drinking water via bowl-type drinkers. The pigs in our study were part of a broader investigation in which other behavioral and physiological indicators related to exploratory behavior were collected. Our study on tail and ear movements ended at 5.5 months of age, and the whole investigation ended one week later, when the pigs were 170 days of age. Concerning the period of the year, the present study started at the beginning of March and finished at the end of May. Pigs were slaughtered in a commercial abattoir after experiencing two different transport conditions for the mentioned broader investigation [31].

### 2.2. Procedure

Thirty-six pigs received one type of environmental enrichment material: twelve pigs were exposed to one of the three types of enrichment material, either straw in a rack, wooden logs or chains. Straw (nonchopped) was continuously provided in a rack (60 × 40 × 80 cm) and not on the floor to avoid large amounts of straw going underneath the slat system and to reduce costs for the farmers. Two wooden logs were hung horizontally, perpendicularly from a chain in each pen (one on the door and another beside the door). The wooden logs (30 × 5 × 5 cm) were near the floor but not touching it. They were made from fresh wood from an ash tree and were replaced after 20 days of use. Two chains (50 cm long) were attached vertically, perpendicularly to the metal fence bars of each pen (one on the door and another beside the door), and as with the wooden logs, they were near the floor but not touching it. Chains are considered of marginal interest according to the EU recommendation [29]. Materials of marginal interest can provide distraction but should not be considered to fulfill the essential needs of the pigs [29]. Thus, such materials do not elicit sufficient exploratory behavior, but this does not mean this type of enrichment is negative for the pigs and that pigs do not interact with it. Additionally, this type of enrichment, despite its low interest, is still widely used on farms. For this reason, this type of enrichment was used for the present study, the aim of which was to investigate tail and ear movements as potential indicators of emotions when pigs were interacting or not interacting with the enrichment, even if the authors acknowledge that the effect of chains may be less positive than that of other enrichment materials. Over three months, behavioral observations were performed once per week by one observer and with the help of one technician: the observer indicated the beginning and ending of each behavior (tail movement and ear movements), and the technician noted the frequencies and durations of each behavior with the help of a stopwatch on a data collection sheet. The interaction with the enrichment materials was scored as a “yes” or “no” for each individual during the two-minute observation. More specifically, focal samplings of each pen were performed every Wednesday morning for 12 weeks, from 9 a.m. to 1 p.m., using a direct observation data collection sheet. The behaviors were described as follows: tail movement, ear movement and interaction with the enrichment. Tail movement was defined as tail swinging in any direction, but mostly from side to side (lateral tail movements) [9,10]; ear movement could be defined as any ear movement or ear posture change, including one or two ears (i.e., changes between ‘front’ and ‘back ear postures’) [9,14,17]; and interaction with the enrichment was defined as any manipulation, exploration, and snout contact with the enrichment material. The frequency and duration of tail movement were analyzed. Tail movement frequency indicates the number of times that a pig starts moving the tail from side to side during the two-minute period. Tail movement duration is expressed as a percentage and means that a pig is moving its tail, and a new movement is considered to begin when it stops the movement for at least two seconds [13,14]. Ear movement is measured only as a frequency because it is considered an event, characterized by behavior patterns of such a short duration that they are difficult to measure over time [32]. Behaviors could overlap and were not considered mutually exclusive. Each animal was observed for two minutes. Before starting the observations, the operators waited for 2 min in front of each pen to allow the animals to acclimate to their presence to minimize any influence on their behavior.

### 2.3. Statistical Analysis

Data analysis was performed using SAS 9.4 software (copyright (c) 2002–2012 by SAS Institute Inc., Cary, NC, USA). The significance threshold was fixed at 5%. The experimental unit was the pen: thus, N = 6. First, differences between the three types of enrichment materials in relation to the three variables (tail movement frequency and duration and ear movement frequency) were analyzed over all 12 weeks. Before analysis, the assumption of the normality of the model residuals was verified with the UNIVARIATE procedure using residual diagnostics plots; homogeneity of variances of the data was verified with the GLM procedure using Levene’s test (using the HOVTEST = LEVENE option in the MEANS statement). As these conditions were met, a General Linear Mixed Model (including the room as a random effect) was carried out using the MIXED PROCEDURE, with multiple comparisons being performed using the LSMEANS statement in the MIXED procedure with the option ADJUST = TUKEY.

The second part of the statistical analysis consisted of a comparison of the variables (tail movement frequency, tail movement duration and ear movement frequency) over all 12 weeks in relation to the use of enrichment regardless of type, which was scored as “high” or “low”: high use of enrichment was considered when three or more animals in the pen interacted with the enrichment material during the 2-min observation of each animal, for a total of six animals in the pen, while low use was considered when fewer than three pigs interacted with the enrichment material during the 2-min observation of each animal. Before analysis, the assumption of the normality of the model residuals was verified as mentioned before. As these conditions were met, a General Linear Mixed Model (including the room as a random effect) was carried out using the MIXED PROCEDURE, with multiple comparisons being performed using the LSMEANS statement in the MIXED procedure with the option ADJUST = TUKEY. See the Supplementary Materials for a detailed description of the full statistical model.

The third part of the analysis consisted of the correlations among the three variables to understand their potential relationships. As the normality of model residuals was verified (UNIVARIATE procedure), correlations among the three variables (tail movement frequency, tail movement duration, and ear movement frequency) for the sum of the 12 weeks were assessed with Pearson’s correlation coefficient using the CORR procedure. According to Martin and Bateson [32], r = 0.4–0.7 is considered to indicate a moderate correlation (substantial relationship), r = 0.7–0.9, a high correlation (marked relationship) and r = 0.9–1.0 a very high correlation (very dependable relationship).

## 3. Results

### 3.1. Comparison of the Variables (Tail Movement Frequency, Tail Movement Duration and Ear Movement Frequency) over all 12 Weeks in Relation to the Type of Enrichment Material

The aim of the study was to investigate potential indicators of emotions (tail movement frequency, tail movement duration and ear movement frequency) in pigs provided with enrichment material at an experimental farm. First, it was necessary to investigate if the different types of enrichment could affect our variables. No significant differences were found among the three types of enrichment materials with regard to tail movement frequency (mean values for enrichment over all 12 weeks: straw rack = 2.19; wooden logs = 2.25; and chains = 2.08; df = 2; F = 0.33; *p* = 0.72), tail movement duration (%) (mean values for enrichment over all 12 weeks: straw rack = 30.67; wooden logs = 28.21; chains = 27.95; df = 2; F = 0.36; *p* = 0.70); or ear movement frequency (mean values for enrichment over all 12 weeks: straw rack = 1.29; wooden logs = 1.38; and chains = 1.40; df = 2; F = 0.11; *p* = 0.90) (Table 1). Therefore, the type of enrichment was not considered in subsequent analyses, and we focused on only whether enrichment materials were being used by the pigs.

### 3.2. Comparison of the Variables (Tail Movement Frequency, Tail Movement Duration and Ear Movement Frequency) over all 12 aweeks in Relation to the Use of Enrichment (Scored as “High” or “Low”) Regardless of Type

A trend, even if not statistically different, was found between the pens with a low or high use of enrichment material for tail movement frequency, which was higher for a high use of enrichment (mean for high use = 2.50; mean for low use = 1.89; df = 1; F = 3.76; *p* = 0.06). A statistically significant difference was found for tail movement duration (%), which was higher for high use of enrichment (mean for high use = 33.15; mean for low use = 25.17; df = 1; F = 4.88; *p* = 0.04). No statistically significant difference was found for ear movement frequency (mean for high use = 1.25; mean for low use = 1.45; df = 1; F = 0.28; *p* = 0.60) (Table 2).

### 3.3. Correlations Among the Three Variables (Tail Movement Frequency, Tail Movement Duration and Ear Movement Frequency) for Data Summed over the 12 Weeks

A positive correlation was found between tail movement frequency and tail movement duration (r = 0.88; *p* = 0.02). No other relevant correlations were found between tail movement frequency and ear movement frequency (r = 0.42; *p* = 0.41) nor between tail movement duration and ear movement frequency (r = 0.03; *p* = 0.95).

## 4. Discussion

The results showed that tail movement duration was significantly higher when the animals interacted more with the enrichment materials than when they with it interacted less during the fattening period. A trend, even if not statistically different, was also found for tail movement frequency, which was also higher with a high use of enrichment. A positive correlation was found between tail movement duration and tail movement frequency. This finding suggests that a high tail movement duration could be an indicator of emotions in fattening pigs with a positive outcome, according to the literature [8,9,13,14,33]. Furthermore, it suggests that a high tail movement frequency could be linked to positive emotions, even if more studies are needed for confirmation, taking into account different total durations of observation and perhaps the ratio between these two parameters. These results suggest that tail movement in pigs could be linked to the use of enrichment materials and therefore to exploratory behavior, which is very important in pigs. The tail movement could also be used to indicate positive emotions, which would indicate positive animal welfare, together with other categories of indicators. Nevertheless, more studies about enrichment materials and different indicators of emotions could be very useful to investigate thoroughly these hypotheses: (i) that enrichment material produces positive emotions to pigs and (ii) that an increase of tail movement indicates positive emotions.

Concerning the relationships among the three parameters (tail movement frequency, tail movement duration and ear movement frequency), the results showed a positive correlation between tail movement frequency and tail movement duration. This finding suggests that an increase in tail movement, either in frequency or duration, could be linked to positive emotions and that both could be used as indicators of emotions with a positive outcome. Previous studies [14] have suggested that, over a ten-minute observation period, a high tail movement duration could be a useful indicator, although the results for frequency were not significant. These results suggest that tail movement could be a valid indicator to assess emotions, together with other categories of indicators. Apart from the duration of tail movement during a set period of time, another feasible measure may be the mean duration of tail movement episodes expressed as a ratio of duration to frequency, but more research is necessary to confirm this notion.

Interestingly, the results of this study also indicated that mini-pigs could be a suitable model of domestic commercial pigs, at least for the parameters observed in the present study, since the results were in accordance with previous studies with mini-pigs. Some studies have examined behavior and welfare in different breeds of mini-pigs [34], and other studies have suggested that welfare indicators of commercial pigs can be used for mini-pigs [35]. Nevertheless, until now, no studies have shown that mini-pigs seem to be a suitable model of domestic commercial pigs, at least for these behavioral parameters.

It is important to remember that all the animals in this study received some type of enrichment material, meaning that all of them could use these materials and that none of the animals were in a nonenriched pen. Tail biting was not observed. The absence of a control group represents a limitation of our study as we had to compare pens only in terms of using more or less enrichment material. Providing enrichment materials to all pigs is mandated by law [28], and many studies have already shown their benefits [19,30,36]. Therefore, in farm conditions, which were used in the present study, no breeders should house their pigs without enrichment materials. The lack of a control group turned into a positive aspect; even though all the animals had the possibility of using enrichment materials, and thus were not in a completely barren environment, they still showed more tail movement when they interacted with the enrichment materials than when they did not. This result suggests that the pigs were more aroused and demonstrated a positive valence [6] during this interaction and that it triggered higher expression of the response behavior (tail movement). In general, the results showed a longer tail movement duration when the animals used the enrichment materials or used them more frequently than when they did not use them or used them less frequently, which suggests that tail movement is linked to positive emotions and therefore to animal welfare. These results are in accordance with the findings of previous studies [9,10,14]. Previous work [13,14] has shown similar results in mini-pigs in an experimental system, and the present study shows that these results can be reproduced at large experimental farms.

Concerning the different types of enrichment material used for the study, although the aim was not to investigate what enrichment was more adequate for the animals, but only if the enrichment, regardless the type, could increase tail movements and decrease ear movements, a preliminary analysis was performed to see if the type of enrichment could affect the main parameters. This preliminary analysis showed no significant difference in tail movement and ear movement in relation to the type of enrichment material. Nevertheless, it is important to clarify that this result could be due to the low sample size or the observation method. These results do not show that chains are equivalent to other enrichment materials such as straw, as it has already been shown that they are not as adequate as other enrichment materials [29].

One limitation of this study was the sampling time: pigs were observed only 2 min per animal per week, because of practical reasons. Accordingly, we analyzed the data by recording if a pig interacted or did not interact with the enrichment (scored as high or low use) but not the amount of time spent interacting with it.

Additionally, we did not confirm that in that case pig enrichment did produce positive emotions to pigs by using other indicators than tail and ear movements: this could also be a limitation. It may have been better to, firstly, corroborate that animals were experiencing positive emotions when interacting with the enrichment using other indicators, and secondly, measure tail and ear movements to obtain more information about these potential indicators. However, this study opens some perspectives for future investigations that would measure thoroughly these potential indicators and the effects of environmental enrichment on emotions.

Another limitation of this study is that the pigs were sometimes lying down or sitting during the observation period and while using the enrichment materials. In these cases, tail movement could not always be measured because the tail could not be observed or it could not move due to direct contact with the floor, both resulting in the behavior being scored as no tail movement. This factor reduced the values for tail movement duration and frequency and could have directly affected the results. This drawback has also been noted by Reimert and colleagues [9]. One possible solution to reduce resting behaviors during observations in future studies could be to have the observer enter the pen before performing the observations to make the pigs stand up, as was performed in the Welfare Quality Protocol [4].

Another topic to thoroughly explore is the fact that all the pigs in the experiment were tail-docked. It would be interesting in the future to compare tail movement in pigs with and without tail-docking, a practice that should be avoided considering the current European legislation of 2008 [37]. Tail-docking may affect tail movements, as well as communication between animals and social interactions [38]. Additionally, the European Union is exerting pressure to completely fulfill the legislation, and a recommendation was published in 2016 [39]. The use of valid indicators such as tail movements could help farmers prevent tail biting.

No significant difference was found for ear movement frequency in relation to whether enrichment materials were used over all 12 weeks. Previous studies have suggested that ear posture changes in pigs are linked to negative emotions [8]. Other studies have consistently suggested that a high ear movement frequency is a direct indicator of negative emotions by showing that the frequency was significantly higher in barren than in enriched conditions [13,14,17]. These latter studies were performed in controlled conditions with mini-pigs, so the aim of the present study was to investigate whether these results could be reproduced in domestic pigs under experimental conditions. For ear movement, we did not obtain the same results as those obtained with mini-pigs. One hypothesis could be that these pigs did not experience negative emotions, or that the observation method (2 min per animal per week) did not allow observation of this behavior or the emission of negative emotions. Another possible explanation for this difference could be the anatomic difference between the ears of mini-pigs (small in proportion to the head) and Landrace × Large White × Pietrain pigs (very large and heavy in proportion of the head). The auricles of pigs are mobile, and they can move to better detect and locate sound [40]. The anatomical structure of auricles as well as their form could vary depending on the breed, which could also affect ear movement [40]. It is possible that large and heavy ears are less mobile than small ones, as suggested by Wei et al. [41]. Another possible explanation is that the rooms where the fattening pigs were housed are noisier than the rooms where mini-pigs were housed, and this could have affected the ear movements. Finally, the two-minute observation period per week may not have been sufficient to obtain significant results when compared to the observation period in previous studies [13,14,17]. In future research, it would be interesting to investigate these possible explanations, increasing the sampling time, as well as to use different breeds of fattening pigs with different types of ears.

## 5. Conclusions

This study provides new perspectives on the evaluation of emotions in farm animals and investigates the suitability of using a high tail movement frequency and duration as indicators of emotions with a positive outcome in pigs. Further research is needed to investigate these potential indicators thoroughly in commercial conditions and the hypothesis that enrichment material could produce positive emotions in pigs. This study also investigated the use of a high ear movement frequency as a possible indicator of emotions with a negative outcome, which was shown in previous controlled studies. However, the present study, performed under experimental farm conditions, was not able to confirm this association. Future studies will be planned to continue investigations in this field of research, as well as to study it in sows, which spend more time on the farm and thus have a higher possibility of encountering situations that impact their emotions. The present results may be a first and preliminary step for improving animal welfare assessments of pigs in different breeding systems when measured in a feasible sample of individuals, and for providing a better understanding of their emotions, an important component of animal welfare.

## Figures and Tables

**Table 1:** Comparison of the variables (tail movement frequency, tail movement duration and ear movement frequency) over all 12 weeks in relation to the type of enrichment material.

Type of Enrichment							
Variable		Straw Rack	Wooden Logs	Chains	df	F	*p*-Value
TMF	N	24	24	24	2	0.33	0.72
	Minimum	0.8	0.3	0.0			
	Maximum	4.0	3.8	3.7			
	Mean	2.3	2.3	2.1			
	SE	0.2	0.2	0.2			
TMD (%)	N	24	24	24	2	0.36	0.70
	Minimum	3.2	1.5	0.0			
	Maximum	58.5	52.9	59.9			
	Mean	30.7	28.2	28.0			
	SE	2.9	2.5	3.0			
EMF	N	24	24	24	2	0.11	0.90
	Minimum	0.0	0.0	0.5			
	Maximum	3.3	4.0	3.8			
	Mean	1.3	1.4	1.4			
	SE	0.2	0.2	0.2			

TMF: Tail movement frequency; TMD: Tail movement duration (%); EMF: Ear movement frequency; N: Number of pens (2 pens for each type of enrichment for 12 weeks); SE: Standard error.

**Table 2:** Comparison of the variables (tail movement frequency, tail movement duration and ear movement frequency) over 12 weeks in relation to the use of enrichment (scored as “high” or “low”).

Use EM						
Variable		High	Low	df	F	*p*-Value
TMF	N	34	38	1	3.76	0.06
	Minimum	0.5	0.0			
	Maximum	3.8	4.0			
	Median	2.7	1.8			
	SE	0.1	0.2			
TMD (%)	N	34	38	1	4.88	0.04
	Minimum	1.5	0.0			
	Maximum	59.9	54.4			
	Median	32.4	25.3			
	SE	13.4	12.6			
EMF	N	34	38	1	0.28	0.60
	Minimum	0.0	0.0			
	Maximum	4.0	3.3			
	Median	1.1	1.3			
	SE	0.2	0.1			

Use EM: Use of enrichment material; High use of enrichment: when three or more pigs in a pen interact with the enrichment material; Low use of enrichment: when fewer than three pigs in a pen interact with the enrichment material; TMF: Tail movement frequency; TMD: Tail movement duration (%); EMF: Ear movement frequency; N High: Number of pens in which the use of enrichment was scored as high; N Low: Number of pens in which the use of enrichment was scored as low; N total = 6 pens × 12 weeks = 72.

## Supplementary Materials

The following are available online at https://www.mdpi.com/2076-2615/9/7/449/s1. File S1: statistical model; Video: Tail movement in tail-docked pigs.
