# Supplementary material for: Are Tail and Ear Movements Indicators of Emotions in Tail-Docked Pigs in Response to Environmental Enrichment?
Source: Animals (Basel). 2019 Jul 16;9(7):449. doi: 10.3390/ani9070449 (PMC6680777; doi:10.3390/ani9070449)
Supplement: Supplementary file 1 [file animals-09-00449-s001.zip › animals-503945-SI/animals-503945-suppl file1.docx]

***Detailed description of the full statistical model.***

The relationship between the variable to be explained (tail movement frequency, duration and ear movement frequency) and explanatory variables (type of enrichment, week and interaction between type of enrichment and week) is defined by the following mathematical equation: $Y= \beta_{1}*X_{1}+\beta_{2}*X_{2}+ \beta_{3}*X_{3}+Z*ϒ+\varepsilon$(E1), where

Y: quantitative variable to be explained, such as “Tail Movement Frequency”, “Tail Movement Duration” or “Ear Movement Frequency”.

Β_j_: coefficients associated with fixed effects.

X_1_: factor “type of enrichment materials” associated with fixed effects.

X_2_: factor “week” associated with fixed effects.

X_3_: interaction between X_1_ and X_2_.

Z: matrix of random effects such as “room” herein.

ϒ: coefficients associated with random effects.

ε: errors associated with each observation.
